# Effect of physical activity promotion on adiponectin, leptin and other inflammatory markers in prediabetes: a systematic review and meta-analysis of randomized controlled trials

**DOI:** 10.1007/s00592-020-01626-1

**Published:** 2020-11-19

**Authors:** Radhika Aditya Jadhav, G. Arun Maiya, Aditi Hombali, Shashikiran Umakanth, K. N. Shivashankar

**Affiliations:** 1 Department of Physiotherapy, Centre for Diabetic Foot Care and Research, Manipal College of Health Professions, Manipal Academy of Higher Education, Manipal, 576104 Karnataka India; 2 Independent Systematic Reviewer, Luton, Bedfordshire UK; 3 Department of Medicine, Melaka Manipal Medical College, Manipal Academy of Higher Education, Manipal, 576104 Karnataka India; 4 Department of Medicine, Kasturba Medical College, Manipal Academy of Higher Education, Manipal, 576104 Karnataka India

**Keywords:** Diabetes, Lifestyle, Inflammation, Cytokines, Adipokines

## Abstract

**Aims:**

Inflammatory stage in prediabetes is associated with increase in level of adipokines and pro-inflammatory cytokines. Physical activity promotion considered as a first-line therapeutic strategy to treat prediabetes. We have conducted the systematic review and meta-analysis to strengthen the evidence on the impact of physical activity promotion on inflammatory markers in prediabetes.

**Methods:**

Studies were identified using electronic search and manual search techniques by choosing keywords for prediabetes, physical activity and inflammatory marker. Randomized controlled trials on individuals diagnosed with prediabetes and provided intervention in the form of physical activity were included in this review. Adiponectin, leptin, C-reactive protein, interleukin-6 and tumour necrosis factor-α were the considered outcome measures.

**Results:**

Our search retrieved 1,688 citations, 31 full-text articles assessed for eligibility of inclusion. Nine studies satisfied the pre-specified criteria for inclusion. Meta-analysis found that physical activity with or without dietary or lifestyle modification reduces level of leptin (MD−2.11 ng/mL, 95% CI −3.81 – −0.42) and interleukin-6 (MD −0.15 pg/mL, 95% CI −0.25–−0.04). It has no effect on level of adiponectin (MD 0.26 µg/mL, 95% CI −0.42– 0.93), C-reactive protein (MD −0.05 mg/L, 95% CI −0.33–0.23) and tumour necrosis factor-α (MD 0.67 pg/mL, 95% CI −2.56–3.89).

**Conclusions:**

This review suggests that physical activity promotion with dietary and lifestyle modification may reduce the level of leptin and interleukin-6 but are uncertain if there is any effect on levels of adiponectin, C-reactive protein and tumour necrosis factor-α in the individuals with prediabetes.

**Electronic supplementary material:**

The online version of this article (10.1007/s00592-020-01626-1) contains supplementary material, which is available to authorized users.

## Introduction

Prediabetes is an intermediate stage of abnormal glucose homeostasis, where blood glucose level is more than a normal range and lesser than the range to confirm type 2 diabetes mellitus (T2DM) [1]. World Health Organization (WHO) and the American Diabetes Association (ADA) have given criteria to define prediabetes [2, 3]. Prediabetes has a high chance of converting into T2DM [4].

Obesity in prediabetes is associated with low-grade chronic systemic inflammation. The level of pro-inflammatory markers increases in prediabetes [5]. Pro-inflammatory state in prediabetes is predominantly because of the increase in insulin resistance [6]. It is considered due to changes in circulating factors released from adipose tissue [7]. Secretions from adipose tissue consist of adipokines and cytokines like C-reactive proteins (CRP), interleukin-6 (IL-6), adiponectin, leptin and tumour necrosis factor-α (TNF-α) [8–10]. Adiponectin and leptin are the adipokines; along with their metabolic property, they are also responsible for inflammation and oxidative stress [11]. High level of leptin activates macrophages and monocytes to produce IL-6 and TNF-α [12]. CRP is an inflammatory protein increase with an increase in inflammation. Thus, like T2DM, prediabetic individuals are also at high risk for development of cardiovascular complications [13].

Physical inactivity is one of the important modifiable risk factors to reduce the burden of prediabetes or type 2 diabetes mellitus (T2DM). Previous literature has reported the impact of physical activity on the risk of T2DM [14, 15]. Physical activity promotion is a first-line therapeutic strategy, which stands before the pharmacological intervention to treat prediabetes [15, 16].

Previous reviews have focused on examining the impact of physical activity intervention on glycaemic parameters and the resulting reduction in aggravation of prediabetes to T2DM [14, 17]. Impact of lifestyle intervention on the level of different inflammatory markers has documented in long-term clinical trials [18–20]. However, there is a diversity in physical activity intervention used and inflammatory markers on which their effects have been seen. Therefore, we have conducted a systematic review and meta-analysis to strengthen the evidence on the impact of physical activity promotion on inflammatory markers in individuals with prediabetes. The main objective of this review is to assess the effect of physical activity promotion on inflammatory markers in individuals with prediabetes.

## Methods

This review is conducted and reported as per the Preferred Reporting Items for Systematic Reviews and Meta-Analysis (PRISMA) checklist [21].

### Information sources

Studies were identified using electronic search as well as manual search techniques. The following databases were searched—PubMed (Medline), Scopus, Web of Science, Cumulative Index to Nursing and Allied Health Literature (CINHAL), Cochrane Central Register of Controlled Trials (CENTRAL) and Embase in December 2019.

### Search strategy

Comprehensive searches were carried out by choosing keywords and subject headings for prediabetes, physical activity and inflammatory marker. Variations in the search terms were identified using truncations and wildcard symbols. The search terms were then combined using Boolean operators. Search filters were used to exclude animal studies and studies published in languages other than English. The identified search terms and the details of the search strategies used in databases have presented in online resource 1. We manually reviewed the references lists of the included studies to identify additional eligible primary studies.

### Study selection

The citations were imported from all the databases to Ryan software [22], and the duplicate citations were identified and removed. The screening process was undertaken in two stages titles: abstract screening and full-text screening. The selection was based on pre-specified review’s inclusion and exclusion criteria. We identified multiple reports from the same study and reported them in online resource 2.

## Eligibility criteria

### Population

Individuals of both gender and age above 20 years, diagnosed with prediabetes by either WHO or ADA criteria, were included. Studies that included individuals diagnosed with type two diabetes mellitus or with normal glucose tolerance were excluded from this review.

### Intervention

We included studies with intervention in the form of physical activity promotion. Physical activity promotion included home-based physical activity or supervised activity session. Any single or multiple forms of physical activity administered in the form of supervised exercises, scheduled activity, weight loss intervention were included. Personal counselling and advice to encourage participation in physical activities along with dietary advice and lifestyle modification were also included. Studies that included other pharmacological intervention along with physical activity were excluded.

### Comparison

Studies that compared physical activity promotion with usual care or no intervention were considered for this review. The usual care intervention group received general information about the benefits of exercises and information about prediabetes and were not included in any form of the physical activity promotion programme.

### Outcome

Studies were included when they measured the following outcome measures—adiponectin, leptin, CRP, IL-6 and TNF-α.

### Types of studies

In this review, we included randomized controlled trials.

### Data collection

Data extraction form was arranged and pilot-tested to extract the study details. Parameters extracted from the included studies were author, year, journal, study design, sample size, age, gender, length of intervention, frequency and intensity of intervention, control and experimental group intervention description, details of the outcome measured and results (online resource 3). For continuous outcomes from randomized controlled trials, we recorded a sample size in each intervention group, mean and standard deviation (SD), mean and standard error (SE), mean and 95% confidence interval (CI) and median and interquartile range (IQR).

### Assessment of risk of bias in included studies

Two authors independently assessed risk of bias of included studies, and the third author resolved disagreements. To assess the risk of bias, we used the Cochrane collaborations tool for risk of bias [23]. Risk of bias assessment was made at the study level. Each included study was assessed on the following key domains: random sequence generation, allocation concealment, blinding, selective outcome reporting, incomplete outcome data and any other risk of bias. Other sources of risk of bias assessment include deviation from study protocol, inappropriate intervention, insensitive instrument, baseline imbalance, contamination and if the study affected by interim results. Each domain was assessed at low, high or unclear risk of bias. The overall risk of bias of each study was assessed at low risk when all the key domains were assessed at low risk, high risk when one or more key domains were assessed at high risk and unclear risk when one or more key domains were assessed at unclear risk of bias.

### Data synthesis

For continuous outcomes, the results were reported as mean difference (MD) with 95% confidence interval (CI). For the studies reporting the median and interquartile range, the values converted to mean and standard deviations by using the appropriate method [24]. In studies with more than two intervention groups, the groups were combined to form a single pairwise comparison using the method set out in the Cochrane handbook [25].

We used Review Manager 5.3 software to perform a meta-analysis. Data available from trails for the outcomes of interest of this review were synthesized using the generic inverse variance method to derive the pooled estimates for continuous variables. Generic inverse variance method was preferred as the included studies reported ‘adjusted’ estimate of treatment effect. Random effects model was used in anticipation of substantial heterogeneity between included studies. The level of heterogeneity was determined by assessing Tau^2^, I^2^ and Chi^2^ statistics. Substantial heterogeneity was considered significant when p < 0.05 in Chi^2^ test and I^2^ > 60% and Tau^2^ is greater than zero. Results with high heterogeneity were interpreted with caution. To explore the heterogeneity, subgroup analysis was not performed as the number of included studies was less than ten.

### Sensitivity analysis

We performed a sensitivity analysis to explore the influence of nonparametric data on effect size by restricting the analysis using four studies reporting parametric data.

## Grade assessment

Two authors have independently assessed the quality of evidence using the GRADE approach with GRADE PRO GDT software [26].

## Results

### Search results

**Fig. 1:**
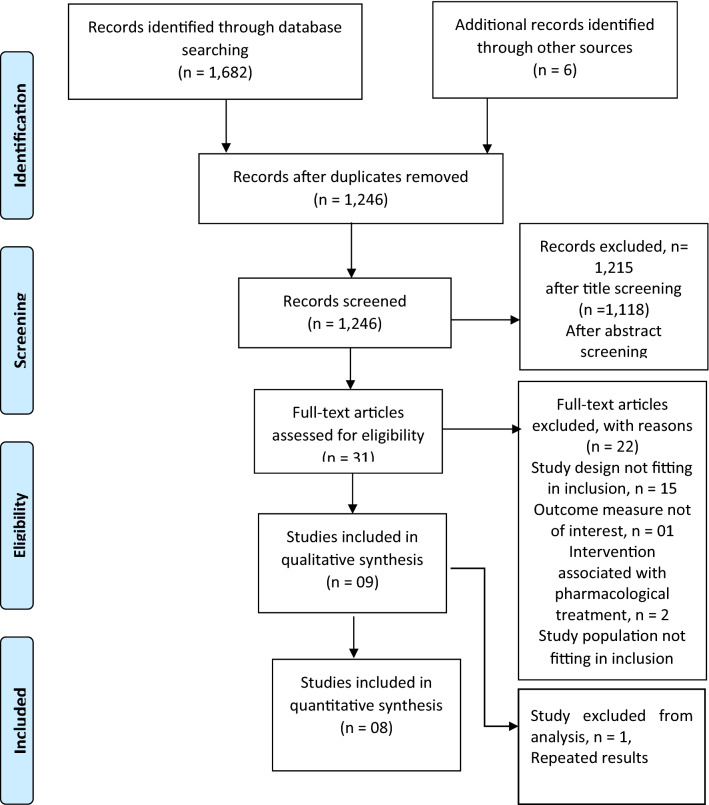
PRISMA flow chart

Our search retrieved 1,688 citations that included electronic and manual search. One thousand two hundred and forty-five (1,245) records screened after duplicate removal. After filtering citations by title screening and abstract screening, 31 full-text articles were assessed for eligibility of inclusion. Nine studies satisfied the pre-specified criteria for inclusion and were included in the review, and eight studies have provided data for meta-analysis. One study was excluded from the meta-analysis as the sample originated from the same study, and the outcome (CRP) was published in two different reports [27]. Details are explained in Fig. 1

### Included studies

#### Study design

For this review, we included nine studies. All included studies were randomized controlled trials (online resource 3).

#### Participants

One thousand nine hundred and six (1,906) participants were included in this systematic review with the age group ranging from 20 to 70 years. All participants were diagnosed by either WHO or ADA.

#### Description of intervention

Eight studies compared the effect of different physical activity (with or without dietary and lifestyle modifications) with usual care on different inflammatory markers in individuals with prediabetes. One study compared the effect of physical activity (with or without dietary and lifestyle modifications) with no intervention on inflammatory markers in individuals with prediabetes.

#### At intervention

Two studies encouraged physical activities along with diet as a part of the study intervention [20, 28]. Two encouraged 140 h of scheduled activities [18, 27]. In one study, physical activity promotion was aimed to give a weight loss programme along with dietary modifications [19]. Other physical activity interventions consisted of moderate to vigorous exercises ≥ 30 min/day [29], walking plus resistance exercise [30], nordic walking [31] and physical activity promotion with and without pedometer [32]. The duration of the intervention varied between included studies. Different durations of intervention were 3 months, 6 months, 1 year and 5 years.

#### At control

Participants received advice on the benefits of exercises and general information about lifestyle, diabetes risk, and information about the consequences and symptoms of impaired glucose tolerance.

### Comparisons

The included studies provided data for two comparisons.

#### Comparison 1

Physical activity with or without dietary or lifestyle modification versus usual care.

Eight studies provided data for this comparison.

#### Comparison 2

Physical activity with or without dietary or lifestyle modification versus no intervention.

Only one study provided data for this comparison.

### Outcome measures

Total nine studies were included to measure the effect of an intervention on adiponectin, leptin, CRP, IL-6 and TNF-α. Five out of nine studies reported adiponectin as one of their outcome measures [19, 20, 28, 31, 32]. Effect of physical activity on leptin was studied in six studies [18–20, 28, 31, 32]. CRP was studied in five [18, 19, 27, 29, 30] and IL-6 in five studies [19, 20, 29–31]. TNF-α was measured in three included studies [19, 20, 31].

### Unit of measurement

We noted that variations in reporting unit of measurement for adiponectin and CRP outcomes. Hence, we converted these values to a standard unit of measurement for each outcome before conducting the meta-analysis.

Adiponectin was reported in different units (ng/ml; ng/l and μg/ml) of measurement in the included studies. We converted the adiponectin values of the studies to μg/ml [19, 20, 32].

CRP values in included studies were reported in mg/l [18, 29, 30]. We converted the CRP values of one study from mg/ml to mg/l [19].

### Combining groups

One study reported leptin values separately for males and females [18]. These subgroup data were combined to form a single group for both intervention and control groups using the method suggested in the Cochrane handbook (Chapter 7, Sect. 7.7.a).

Three studies compared more than two intervention groups [30–32]. Two of the intervention groups from these studies were combined to form a single intervention group using the method suggested in the Cochrane handbook (Chapter 7, Sect. 7.7.a).

### Risk of bias of included studies

Among the nine included studies in this review, two studies [20, 30] had a low overall risk of bias assessment. Four studies [19, 28, 29, 31] had a high overall risk of bias, and three studies [18, 27, 32] had an unclear overall risk of bias.

We assessed seven studies at low risk of selection bias [18–20, 28–31] as they adequately reported sequence generation for recruiting participants. Two studies [27, 32] were assessed at unclear risk of bias. Two studies [20, 30] adequately reported the method used for allocation of participants and were assessed at low risk of bias. Five studies [18, 19, 27–29, 32] were assessed at unclear risk and one study [31] was assessed at high risk as the study did not effectively conceal the allocation of participants to intervention groups.

Due to the type of intervention, blinding of participants and professionals providing treatment was not feasible; hence, this domain was assessed at low risk of bias. Similarly, we assessed blinding of outcome assessors at low risk of bias as the numerical values of the outcome of interest were obtained from independent laboratory tests and thus would not influence the intervention effect estimates.

For reporting bias, four studies [20, 27, 30, 31] had a published protocol and there was no indication of selective reporting and hence were assessed at low risk. Three studies [18, 28, 32] were assessed at unclear risk, as there was no registered protocol available. We judged two studies [19, 29] at high risk for reporting outcomes of interest of this review that were not included as a part of the trial protocol.

For incomplete outcome data, we assessed two studies [28, 29] at high risk as the dropout rate was more than 20%. We did not identify any other potential sources of bias from the included studies (online resource 4).

### Effects of intervention

#### Comparison 1: physical activity with or without dietary or lifestyle modification versus control

Seven studies contributed to this comparison [18–20, 28–31].

### Adiponectin (µg/ml)

**Fig. 2:**
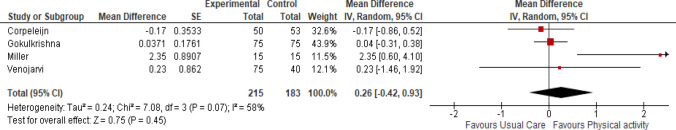
Analysis 1.1: effect of physical activity with or without dietary or lifestyle modification versus usual care on adiponectin

Four studies measured adiponectin levels after three months [31], four months [20] and one year [19, 28] of intervention. Physical activity did not have any effect on adiponectin levels (Fig. 2: Analysis 1.1) (mean difference (MD) 0.26 µg/ml, 95% CI -0.42 to 0.93; participants = 398; four studies, I^2^ = 58%, certainty of the evidence is very low).

The sensitivity analysis was conducted by excluding one study [28]. The results suggest the adiponectin levels may increase in the physical activity intervention group as compared to the usual care group (MD 0.69 µg/ml, 95% CI -0.62 to 2.00; participants = 295; three studies, I^2^ = 69%).

#### Leptin (ng/ml)

**Fig. 3:**
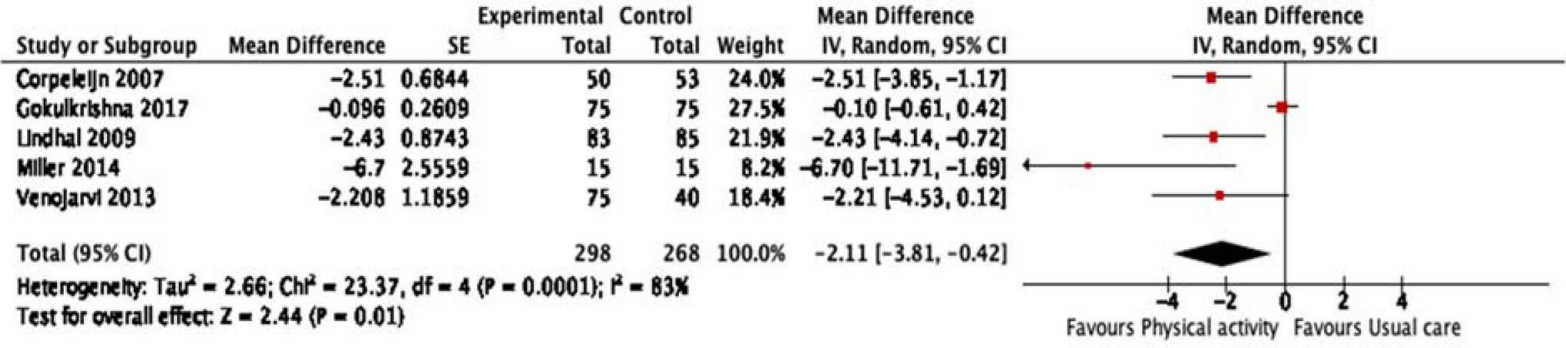
Analysis 1.2: effect of physical activity with or without dietary or lifestyle modification versus usual care on leptin

Five studies measured leptin levels after three months [31], four months [20], one year [19, 28] and one study after five years [18] of intervention. Physical activity reduced the leptin levels in prediabetic individuals (Fig. 3: Analysis 1.2) (MD -2.11 ng/ml, 95% CI -3.81 to -0.42; participants = 566; five studies, I = 83%, certainty of the evidence is very low).

The sensitivity analysis was conducted by excluding one study [28]. We observed that the direction of effect of intervention did not change for leptin (MD -2.05 ng/ml, 95% CI -4.11 to -0.01; participants = 463; four studies, I^2^ = 80%).

#### C-reactive protein (CRP) (mg/l)

**Fig. 4:**
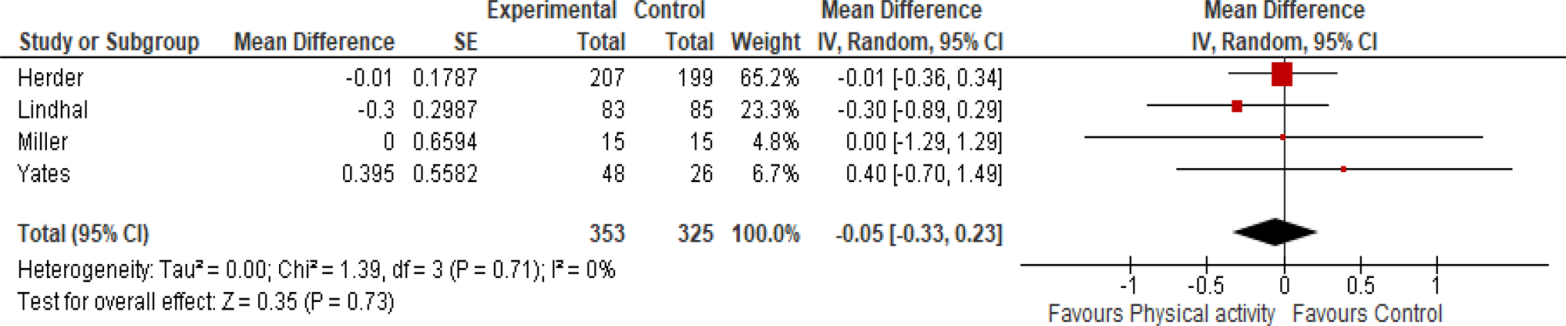
Analysis 1.3: effect of physical activity with or without dietary or lifestyle modification versus usual care on CRP

Four studies measured CRP levels after one year [19, 29, 30] and one study after five years [18] of intervention. Physical activity did not have any effect on the CRP levels in prediabetic individuals (Fig. 4: Analysis 1.3) (MD -0.05 mg/l, 95% CI -0.33 to 0.23, four studies, 678 participants, I^2^ = 0%, certainty of the evidence is low).

The sensitivity analysis was conducted by excluding two studies [29, 30]. We observed that the direction of effect of intervention did not change for CRP (MD -0.25 mg/l, 95% CI -0.78 to 0.28, two studies, 198 participants, I^2^ = 0%).

#### Interleukin- 6 (IL-6) (pg/ml)

**Fig. 5:**
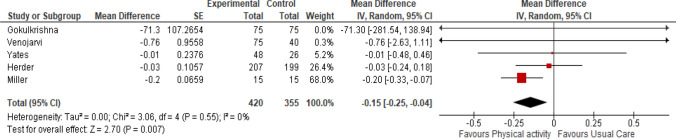
Analysis 1.4: effect of physical activity with or without dietary or lifestyle modification versus usual care on IL-6

Five studies measured IL-6 after one year [19, 29, 30], four months [20] and one study after three months [31] of intervention. Physical activity reduced the interleukin-6 levels in prediabetic individuals (Fig. 5: Analysis 1.4) (MD -0.15 pg/ml, 95% CI -0.25 to -0.04; participants = 775; five studies, I^2^ = 0%, certainty of the evidence is low).

The sensitivity analysis was conducted by excluding two studies [29, 30]. We observed that the direction of effect of intervention did not change for IL-6 (MD -0.20 pg/ml, 95% CI -0.33 to -0.07; participants = 295; three studies, I^2^ = 0%).

#### Tumour necrosis factor-α (TNF-α) (pg/ml)

**Fig. 6:**
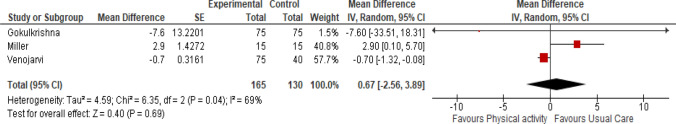
Analysis 1.5: effect of physical activity with or without dietary or lifestyle modification versus usual care on TNF-α

Three studies measured TNF-α after three months [31], four months [20] and one year [19] of intervention. Physical activity did not have any effect on TNF-α in prediabetic individuals (Fig. 6: Analysis 1.5) (MD 0.67 pg/ml, 95% CI -2.56 to 3.89; participants = 295; three studies, I^2^ = 69%, certainty of the evidence is very low).

#### Comparison 2: physical activity with or without dietary or lifestyle modification versus no intervention

Only one study provided data for this comparison and provided two outcomes: adiponectin and leptin [32].

#### Adiponectin (µg/ml)

Physical activity intervention did not have any effect on adiponectin levels in prediabetic individuals (mean difference (MD) 1.30 μg/ml, 95% CI -0.57 to 3.17, one study, 61 participants, certainty of the evidence is very low).

#### Leptin (ng/ml)

Physical activity intervention reduced the leptin levels in prediabetic individuals (MD -2.08 ng/ml, 95% CI -3.87 to 0.29, one study, 61 participants, certainty of the evidence is very low).

## Discussion

In this review, we investigated the effect of physical activity promotion programme on inflammatory markers in individuals with prediabetes. We included nine trials with total 1,906 participants. The meta-analysis was performed to compare the effect of physical activity with or without dietary or lifestyle modification versus usual care (comparison 1) and physical activity with or without dietary or lifestyle modification versus no intervention (comparison 2) on inflammatory markers adiponectin, leptin, CRP, IL-6 and TNF-α. Out of the nine studies, we included eight studies for meta-analysis; seven studies were included in the first comparison, and one study was included in the second comparison. In the first comparison, the physical activity intervention administered along with or without dietary or lifestyle modification may reduce the level of leptin and IL-6 in individuals with prediabetes. On the other hand, we are uncertain if the intervention would affect the level of adiponectin, CRP and TNF-α. In the second comparison, only one study provided data and we found that physical activity with or without dietary or lifestyle modification, when compared to the group that did not receive any form of intervention, had no effect on the level of adiponectin, but it reduces the level of leptin in individuals with prediabetes.

The grade assessment for certainty of evidence was very low for adiponectin, CRP and TNF- α (no effect); very low for leptin and low for IL-6 (favours physical activity) in the first comparison. The studies were downgraded for certainty of evidence mainly due to inconsistency, imprecision, risk of bias. For the second comparison, only one study reported adiponectin and leptin outcomes (summary of findings presented in online resource 5).

As per Cochrane risk of bias tool, we assessed trials as low, high or unclear risk. We contacted all the authors of the included trial to confirm our judgement. Nearly 25% of the studies had insufficient information on sequence generation, and about half of the studies had no information on methods used to conceal allocation. Less than 15% of the studies were assessed at high risk for inadequate allocation concealment. High attrition rates were reported in less than 25% of the studies. Majority of the included studies had inadequate reporting of outcomes of interest.

We have identified previously published systematic reviews and meta-analysis that evaluated the effect of physical activity on various inflammatory markers. Becic et al. 2018 included individuals with diabetes and prediabetes and found that physical exercise reduced leptin levels and increased adiponectin levels [33]. Yu N et al. 2016 evaluated the effect of exercise on leptin and adiponectin in overweight and obese individuals. They also found that exercise reduced leptin levels and increased adiponectin levels [34]. Serico et al. 2018 investigated that physical exercise without any concomitant dietary intervention improved leptin, adiponectin and IL-6 in children with obesity [35]. Anche et al. 2020 investigated the effect of lifestyle modification and physical activity promotion on leptin in individuals with metabolic syndrome included both randomized and non-randomized trials [36]. Zeng et al. 2019 assessed the effect of aerobic exercises on inflammatory markers in healthy middle-aged and elderly adults suggested a positive effect of an intervention on CRP, TNF-α and IL- 6 [37]. Our review differed from the previous review in terms of population and the interventions. We included only prediabetes population as per ADA or WHO criteria between the age group of 20 to 70 years, and the intervention focused mainly on the physical activity promotion programme administered along with or without dietary or lifestyle modifications. Hence, the evidence of this review does not apply to the paediatric population or normoglycaemic adults.

Effect of exercise on pro-inflammatory cytokines has been inconsistent. The different reasons include health status, age, sex, disease, type of exercise, duration and intensity of exercise [38]. Changes in the level of inflammatory markers due to physical activity promotion are directly proportional to the mode, duration and intensity of an activity. Combined exercise has a better anti-inflammatory effect than aerobic or resistance exercise alone [39]. There was dissimilarity in the type of interventions in terms of its mode, duration, type of monitoring and intensity administered in the studies included in this review. This could be one of the reasons for finding the different results on the included outcome measures. However, it should also be noted that there were very few studies that contributed data to each outcome and the included studies had small sample size which could have shown a greater effect of the intervention and were possibly underpowered to detect the desired effect of the intervention on multiple outcomes. Sensitivity analysis of adiponectin, CRP and TNF-α suggests that the intervention did have a positive effect on adiponectin levels, while the other outcomes did not show any change. Due to the limited number of studies, we could not perform a sensitivity analysis by excluding studies with a high risk of bias and subgroup analysis to explore the heterogeneity.

We found that physical activity promotion with or without dietary or lifestyle modification did not affect adiponectin level, however showed a positive effect on leptin in individuals with prediabetes. There exists a dose-response relationship of adiponectin and leptin with weight loss in human participants indicating that mild weight loss up to 5% reduces the leptin level but to make a change in the level of adiponectin weight reduction should be more than 10% [40]. Prediabetes stage is associated with the increase in the level of inflammatory markers, that is, CRP, IL-6 and TNF-α [9, 10, 13, 41]. IL-6 promotes the expression of leptin on mRNA and inhibits the expression of adiponectin. This explains the impact of physical activity on adiponectin and leptin by reducing IL-6 [42]. Weight reduction causes a reduction in the size of adipocyte and reduction in the secretions of inflammatory cytokines. The concentration of IL-6 correlates with the size of adipocyte [43]. However, secretion of TNF-α is independent of the size of adipocyte [44, 45]. The literature on cytokine production has also proposed that IL-6 suppresses the production of TNF-α [46].

## Limitations

This review has few limitations; we only considered the published literature and English language. Grey literature or research registry was not incorporated into the search. However, we could not assess publication bias using a funnel plot, as we had less than ten studies qualified for inclusion. There are very few studies included in this review under each outcome and comparison to provide recommendations for clinical applicability.

### Implication for future research

Physical activity promotion may reduce the inflammation in the individuals with prediabetes; however, level of its evidence is very low; also there is a dearth of literature on minimally clinical important difference (MCID) value for considered outcome measures. Further trials are needed to focus on this specific question with robust sample size; methodological rigour and long-term follow-up to estimate the effect of physical activity promotion on inflammatory markers in individuals with prediabetes.

## Conclusion

This review suggests that physical activity promotion programme may reduce the level of leptin and IL-6, but it is uncertain whether there is any effect on the level of adiponectin, CRP and TNF-α in individuals with prediabetes.

## Electronic supplementary material

Below is the link to the electronic supplementary material.

Supplementary material 1 (DOCX 14 kb)

Supplementary material 2 (DOCX 18 kb)

Supplementary material 3 (DOCX 17  kb)

Supplementary material 4 (DOCX 28 kb)

Supplementary material 5 (DOCX 20 kb)

## Data Availability

All the data generated and analysed during this review have been provided in article and in supplementary files.

## Abbreviations

T2DM: Type 2 diabetes mellitus
ADA: American Diabetes Association
WHO: World Health Organization
IL-6: Interleukin 6
CRP: C-reactive protein
TNF-α: Tumour necrosis factor-α
MCID: Minimally clinical important difference
PA: Physical activity
