# Supplementary material for: Effect of physical activity promotion on adiponectin, leptin and other inflammatory markers in prediabetes: a systematic review and meta-analysis of randomized controlled trials
Source: Acta Diabetol. 2020 Nov 19;58(4):419–29. doi: 10.1007/s00592-020-01626-1 (PMC8053655; doi:10.1007/s00592-020-01626-1)
Supplement: Supplementary file 1 — Supplementary material 1 (DOCX 14 kb) [file 592_2020_1626_MOESM1_ESM.docx]

**Effect of physical activity promotion on adiponectin, leptin and other inflammatory markers in prediabetes – A systematic review and meta-analysis of randomized controlled trials**

**Journal: Acta Diabetologica**

*Authors: Radhika Aditya Jadhav, Dr. Arun G Maiya*, Aditi Hombali, Dr. Shashikiran U, Dr. Shivashankar K N,*

*Corresponding author: Dr. Arun G Maiya**

*Centre for diabetic foot care and research, Department of Physiotherapy, Manipal College of Health Professions, Manipal academy of Higher Education, Manipal- 576104, Karnataka, India*

*Email: arun.maiya@manipal.edu*

**Electronic Supplementary file 1: Search Strategy**

Population: “prediabet*” or “pre diabet*” or “intermediate hyperglyc?emi*” or “impaired fasting ADJ2 glucose” or “IFG” or “impaired FPG” or “glucose intolerance” or “impaired glucose ADJ tolerance” or “IGT” or “risk ADJ3 type 2 or type II or diabetes or T2D* or NIDDM” or “borderline diabetes*”

Intervention: “physical activit*” or “motor activit*” or “exercis*” or “resistance training*” or “aerobic execis*” or “lifestyle or life style ADJ3 intervention? or change* or modif* or program or programme” or “fitness” or “yoga” or “sport*”

Outcome: “biomarker*” or “marker*” or “cytokine*” or “TNF‐a” or “tumour necrosis factor* alfa” or “inflammatory marker*” or “interleukin‐6” or “IL-6” or “adiponectin” or “leptin” or “CRP” or “C reactive protein”
