# Supplementary material for: Effect of physical activity promotion on adiponectin, leptin and other inflammatory markers in prediabetes: a systematic review and meta-analysis of randomized controlled trials
Source: Acta Diabetol. 2020 Nov 19;58(4):419–29. doi: 10.1007/s00592-020-01626-1 (PMC8053655; doi:10.1007/s00592-020-01626-1)
Supplement: Supplementary file 2 — Supplementary material 2 (DOCX 18 kb) [file 592_2020_1626_MOESM2_ESM.docx]

**Effect of physical activity promotion on adiponectin, leptin and other inflammatory markers in prediabetes – A systematic review and meta-analysis of randomized controlled trials**

**Journal: Acta Diabetologica**

*Authors: Radhika Aditya Jadhav, Dr. Arun G Maiya*, Aditi Hombali, Dr. Shashikiran U, Dr. Shivashankar K N,*

*Corresponding author: Dr. Arun G Maiya**

*Centre for diabetic foot care and research, Department of Physiotherapy, Manipal College of Health Professions, Manipal academy of Higher Education, Manipal- 576104, Karnataka, India*

*Email:* [*arun.maiya@manipal.edu*](mailto:arun.maiya@manipal.edu)

**Electronic Supplementary file 2: Multiple reports of included trial**

| ***Study on Lifestyle-intervention and Impaired glucose tolerance Maastricht (SLIM):***   1. Mensink M, Blaak EE, Corpeleijn E, Saris WH, De Bruin TW, Feskens EJ. Lifestyle intervention according to general recommendations improves glucose tolerance. *Obes Res*. 2003;11(12):1588-1596. doi:10.1038/oby.2003.211 2. Mensink M, Corpeleijn E, Feskens EJM, et al. Study on lifestyle-intervention and impaired glucose tolerance Maastricht (SLIM): Design and screening results. *Diabetes Res Clin Pract*. 2003;61(1):49-58. doi:10.1016/S0168-8227(03)00067-6 3. *Corpeleijn E, Feskens EJM, Jansen EHJM, Mensink M, Saris WHM, Blaak EE. Lifestyle intervention and adipokine levels in subjects at high risk for type 2 diabetes: The Study on Lifestyle intervention and Impaired glucose tolerance Maastricht (SLIM). *Diabetes Care*. 2007;30(12):3125-3127. doi:10.2337/dc07-0457 |
| --- |
| ***The Diabetes Community Lifestyle Improvement Program (D-CLIP) trial :***   1. Weber MB, Ranjani H, Meyers GC, Mohan V, Narayan KMV. A model of translational research for diabetes prevention in low and middle-income countries: The Diabetes Community Lifestyle Improvement Program (D-CLIP) trial. *Prim Care Diabetes*. 2012;6(1):3-9. doi:10.1016/j.pcd.2011.04.005 2. Weber MB, Ranjani H, Staimez LR, et al. The stepwise approach to diabetes prevention: Results from the D-CLIP randomized controlled trial. *Diabetes Care*. 2016;39(10):1760-1767. doi:10.2337/dc16-1241 3. *Gokulakrishnan K, Ranjani H, Weber MB, et al. Effect of lifestyle improvement program on the biomarkers of adiposity, inflammation and gut hormones in overweight/obese Asian Indians with prediabetes. *Acta Diabetol*. 2017;54(9):843-852. doi:10.1007/s00592-017-1015-9 |
| ***Diabetes Prevention Study (DPS):***   1. Eriksson J, Lindström J, Valle T, et al. Prevention of Type II diabetes in subjects with impaired glucose tolerance: The Diabetes Prevention Study (DPS) in Finland. Study design and 1-year interim report on the feasibility of the lifestyle intervention programme. *Diabetologia*. 1999;42(7):793-801. doi:10.1007/s001250051229 2. *Herder C, Peltonen M, Koenig W, et al. Anti-inflammatory effect of lifestyle changes in the Finnish Diabetes Prevention Study. *Diabetologia*. 2009;52(3):433-442. doi:10.1007/s00125-008-1243-1 |
| ***Pre-diabetes Risk Education and Physical Activity Recommendation and Encouragement (PREPARE) programme study:***   1. Yates T, Davies M, Gorely T, Bull F, Khunti K. Rationale, design and baseline data from the Pre-diabetes Risk Education and Physical Activity Recommendation and Encouragement (PREPARE) programme study: A randomized controlled trial. *Patient Educ Couns*. 2008;73(2):264-271. doi:10.1016/j.pec.2008.06.010 2. *Yates T, Davies MJ, Gorely T, et al. The effect of increased ambulatory activity on markers of chronic low-grade inflammation: Evidence from the PREPARE programme randomized controlled trial. *Diabet Med*. 2010;27(11):1256-1263. doi:10.1111/j.1464-5491.2010.03091 |
| ***Healthy Living Partnerships to Prevent Diabetes (HELP PD): Design and Methods:***   1. Jeffrey A. Katula, Mara Z. Vitolins, Erica L. Rosenberger, Blackwell, Mark A. Espeland, Michael S. Lawlor and David C. Goff, Healthy Living Partnerships to Prevent Diabetes (HELP PD): Design and Methods. *Contemp Clin Trials*. 2011;31(1):1-19. doi:10.1016/j.cct.2009.09.002.Healthy 2. *Miller GD, Isom S, Morgan TM, et al. Effects of a community-based weight loss intervention on adipose tissue circulating factors. *Diabetes Metab Syndr*. 2015;8(4):205-211. doi:10.1016/j.dsx.2014.09.003.Effects |
| ***Va¨sterbotten Intervention Programme (VIP):***   1. Weinehall L. 1997. Partnership for health. On the role of primary health care in a community intervention programme. Umea° University Medical Dissertations 1997, New series No 531. 2. Lindahl B, Nilsson TK, Jansson JH, Asplund K, Hallmans G. 1999. Improved fibrinolysis by intense lifestyle intervention. A randomized trial in subjects with impaired glucose tolerance. Journal of Internal Medicine 246:105_112. 3. *Lindahl B, Söderberg S, Widman L, et al. A randomized lifestyle intervention with 5-year follow-up in subjects with impaired glucose tolerance: Pronounced short-term impact but long-term adherence problems. *Scand J Public Health*. 2009;37(4):434-442. doi:10.1177/1403494808101373 4. *Andersson, J., Boman, K., Jansson, J. H., Nilsson, T. K., & Lindahl, B. (2008). Effect of intensive lifestyle intervention on C-reactive protein in subjects with impaired glucose tolerance and obesity. Results from a randomized controlled trial with 5-year follow-up. *Biomarkers, 13*(7), 671-679. doi:10.1080/13547500802661266 |

**Included trial*
