# Supplementary material for: Effect of physical activity promotion on adiponectin, leptin and other inflammatory markers in prediabetes: a systematic review and meta-analysis of randomized controlled trials
Source: Acta Diabetol. 2020 Nov 19;58(4):419–29. doi: 10.1007/s00592-020-01626-1 (PMC8053655; doi:10.1007/s00592-020-01626-1)
Supplement: Supplementary file 3 — Supplementary material 3 (DOCX 17 kb) [file 592_2020_1626_MOESM3_ESM.docx]

**Effect of physical activity promotion on adiponectin, leptin and other inflammatory markers in prediabetes – A systematic review and meta-analysis of randomized controlled trials**

**Journal: Acta Diabetologica**

*Authors: Radhika Aditya Jadhav, Dr. Arun G Maiya*, Aditi Hombali, Dr. Shashikiran U, Dr. Shivashankar K N,*

*Corresponding author: Dr. Arun G Maiya**

*Centre for diabetic foot care and research, Department of Physiotherapy, Manipal College of Health Professions, Manipal academy of Higher Education, Manipal- 576104, Karnataka, India*

*Email:* [*arun.maiya@manipal.edu*](mailto:arun.maiya@manipal.edu)

**Electronic Supplementary file 3: Table 1: Characteristics of the included studies**

| Sr. No. | Author | Year | Journal | Study Design | Age group | Sample size | Intervention | | Control | Duration |
| --- | --- | --- | --- | --- | --- | --- | --- | --- | --- | --- |
| 1. | Corpeleijn E et al, | 2007 | Diabetes Care | RCT | 40 to 60 years | 103 | Dietary recommendation along with PA simulation as per ACSM recommendation. Individual advice and subjects were encouraged to participate in exercise programme. | | Informed about beneficial effect of healthy diet, PA and weight loss. No individual advice. | 1 year |
| 2. | Andersson J et al, | 2008 | Biomarkers | RCT | 30 to 60 years | 200 | 140 Hrs. of scheduled activities or low to moderate intensity that included brisk walking, gymnastics, cycling, swimming | | Oral and written advice on lifestyle intervention on impaired glucose tolerance and obesity. | 1Year |
| 3. | Miller G D et al, | 2014 | Diabetes Metabolic Syndrome | RCT | 45 to 70 years | 30 | Lifestyle weight loss intervention programme included healthy eating and increase physical activities | | Dietician session and newsletter to support weight loss. | 1 year |
| 4. | Herder C et al, | 2009 | Diabetologia | RCT | 40 to 65 years | 522 | Moderate to vigorous exercises ≥ 30 min/day, Advice to increase overall physical activity and dietary advice | | General information about lifestyle and diabetes risk. | 1 year |
| 5. | Lindahl B et al, | 2009 | Scandinavian Journal of Public Health | RCT | 40 to 60 years | 194 | 140 Hrs. of scheduled activities including aerobic physical activity of moderate intensity and diet recommendation. | | Counselling session including oral and written advice. | 1 year |
| 6. | Yates T et al, | 2010 | Diabetic Medicine | RCT | 47 to 63 | 74 | 180 minutes of Physical activity recommendation and encouragement programme along with pedometer | Physical activity recommendation and encouragement programme without pedometer but asked to set a time-based goal. | Information about consequences and symptoms of impaired glucose tolerance. | 1 year |
| 7. | Liu Y et al, | 2017 | Biomedical Research | RCT | 45 to 55 years | 61 | Walking exercise for 60 min. 4 times per week with intensity 60%-70% HRmax. | Walking plus resistance exercise included walking for 20 min. and resistance exercises for 30 min. | No participation in exercises. | 6Months |
| 8. | Venojarvi M et al, 2013 | 2013 | Annals of Medicine | RCT | 40 to 65 | 144 | Nordic walking (60 minutes session, 3times per week) including warm up, cool down and walking with the help of pole with the intensity of 55 to 75% of heart rate reserve. | Resistance training session (60 minutes 3 times per week) including strength and power exercises of upper limb, lower limb and trunk with load 5RM | Advice regarding health benefits of exercises. | 3Months |
| 9. | Gokulkrishnan K et al, | 2017 | Acta Diabetol | RCT | 20 to 65 years | 578 | Intensive lifestyle intervention including exercises and diet. | | Single visit to physician, dietician and fitness trainer followed by common group education class on diabetes prevention. | 4 months |

HRmax- heart rate maximum; ACSM- American College of Sports Medicine ; RCT- Randomized controlled trial, PA - Physical Activity

**Table 2: outcomes and results of included trials**

| Sr. No. | Author | Year | Outcomes | Results |
| --- | --- | --- | --- | --- |
| 1. | Corpeleijn E et al, | 2007 | Adiponectin, Leptin | Lifestyle intervention reduced plasma leptin concentrations (_14.2%) in IGT subjects but did not alter plasma adiponectin (-0.3%), despite marked improvements in glucose tolerance and insulin resistance. |
| 2. | Andersson J et al, | 2008 | CRP | At the 1-year follow-up, there was no difference in CRP between the two treatment groups. |
| 3. | Herder C et al, | 2009 | CRP, IL-6 | Lifestyle intervention reduced circulating levels of CRP (p<0.001) and IL-6 (p=0.060). |
| 4. | Lindahl B et al, | 2009 | Leptin, CRP | At 1-year follow-up, in the intensive intervention group, a 70% decrease of progress to type 2 diabetes. |
| 5. | Yates T et al, | 2010 | CRP, IL-6 | There was no significant change in markers of chronic low-grade inflammation. CRP (P=0.73), IL-6 (P=0.79) |
| 6. | Venojarvi M et al, | 2013 | Adiponectin, Leptin, IL-6, TNF-α | Plasma leptin concentration decreased in the Nordic walking group ( *P* _ 0.001) No significant change in adiponectin, IL-6 and TNFα concentration between the groups. |
| 7. | Miller G D et al, | 2014 | Adiponectin, Leptin, CRP, IL-6, TNF-α | These findings suggest adipose tissue generated mediators of cardiovascular risk can be improved with weight loss. There was no difference for CRP between the groups but Adiponetin (p = 0.02), Leptin (p = 0.02), IL-6 and TNF-α (p = 0.01) were significantly improved in intervention group. |
| 8. | Liu Y et al, | 2017 | Adiponectin, Leptin | Exercise intervention decreased leptin significantly, but increased adiponectin. (p<0.05) but no difference in two exercise groups. |
| 9. | Gokulkrishnan K et al, | 2017 | Adiponectin, Leptin, IL-6, TNF-α | Participants in the intervention group showed significant reductions (p<0.001) in plasma levels of leptin (17.6%), TNFa (35%), IL-6 (33.3%) and increased levels of adiponectin (33.1) |
