# Supplementary material for: Effect of physical activity promotion on adiponectin, leptin and other inflammatory markers in prediabetes: a systematic review and meta-analysis of randomized controlled trials
Source: Acta Diabetol. 2020 Nov 19;58(4):419–29. doi: 10.1007/s00592-020-01626-1 (PMC8053655; doi:10.1007/s00592-020-01626-1)
Supplement: Supplementary file 4 — Supplementary material 4 (DOCX 28 kb) [file 592_2020_1626_MOESM4_ESM.docx]

**Effect of physical activity promotion on adiponectin, leptin and other inflammatory markers in prediabetes – A systematic review and meta-analysis of randomized controlled trials**

**Journal: Acta Diabetologica**

*Authors: Radhika Aditya Jadhav, Dr. Arun G Maiya*, Aditi Hombali, Dr. Shashikiran U, Dr. Shivashankar K N,*

*Corresponding author: Dr. Arun G Maiya**

*Centre for diabetic foot care and research, Department of Physiotherapy, Manipal College of Health Professions, Manipal academy of Higher Education, Manipal- 576104, Karnataka, India*

*Email:* [*arun.maiya@manipal.edu*](mailto:arun.maiya@manipal.edu)

**Electronic Supplementary file 4**: **Summary of risk of bias assessment**


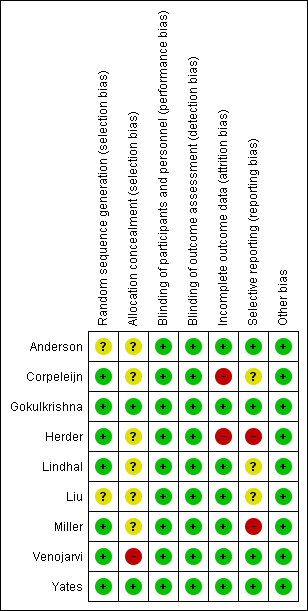


**Fig.1:** Review authors' judgements about each risk of bias item for each included study


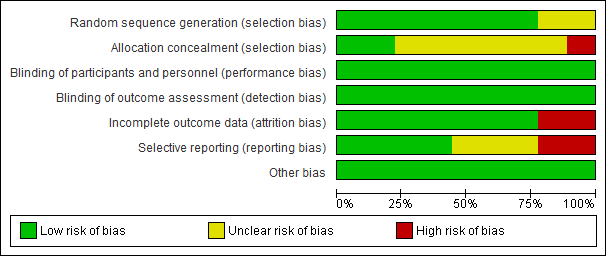


Fig.2: Risk of bias graph: review authors' judgements about each risk of bias item presented as percentages across all included studies.
