# Supplementary material for: Effect of physical activity promotion on adiponectin, leptin and other inflammatory markers in prediabetes: a systematic review and meta-analysis of randomized controlled trials
Source: Acta Diabetol. 2020 Nov 19;58(4):419–29. doi: 10.1007/s00592-020-01626-1 (PMC8053655; doi:10.1007/s00592-020-01626-1)
Supplement: Supplementary file 5 — Supplementary material 5 (DOCX 20 kb) [file 592_2020_1626_MOESM5_ESM.docx]

**Effect of physical activity promotion on adiponectin, leptin and other inflammatory markers in prediabetes – A systematic review and meta-analysis of randomized controlled trials**

**Journal: Acta Diabetologica**

*Authors: Radhika Aditya Jadhav, Dr. Arun G Maiya*, Aditi Hombali, Dr. Shashikiran U, Dr. Shivashankar K N,*

*Corresponding author: Dr. Arun G Maiya**

*Centre for diabetic foot care and research, Department of Physiotherapy, Manipal College of Health Professions, Manipal academy of Higher Education, Manipal- 576104, Karnataka, India*

*Email:* [*arun.maiya@manipal.edu*](mailto:arun.maiya@manipal.edu)

**Electronic Supplementary file 5:** Grade analysis

Summary of findings table 1: Physical activity with or without dietary or lifestyle modification compared to usual care for Prediabetes (comparison 1)

| **Physical activity with or without dietary or lifestyle modification compared to usual care for Prediabetes** | | | | | | |
| --- | --- | --- | --- | --- | --- | --- |
| **Patient or population**: Prediabetes  **Setting**: Netherlands, United States, Finland, Sweden, United Kingdom, India  **Intervention**: Physical activity with or without dietary or lifestyle modification  **Comparison**: usual care | | | | | | |
| Outcomes | **Anticipated absolute effects^*^** (95% CI) | | Relative effect (95% CI) | № of participants  (studies) | Certainty of the evidence (GRADE) | Comments |
|  | **Risk with usual care** | **Risk with Physical activity with or without dietary or lifestyle modification** |  |  |  |  |
| Adiponectin | The mean adiponectin (µg/ml) in the intervention group was **0**.26 higher (0.41 lower to 0.93 higher) | | - | 398 (4 RCTs) | ⨁◯◯◯ VERY LOW ^a,b,c^ | Included studies: Corpeleijn 2007; Gokulkrishna 2017; Miller 2014; Venojarvi 2013 |
| Leptin | The mean leptin (ng/ml) in the intervention group was 2.11 lower (3.81 lower to 0.42 lower) | | - | 566 (5 RCTs) | ⨁◯◯◯ VERY LOW ^a,b,d^ | Included studies: Corpeleijn 2007; Gokulkrishna 2017; Lindhal 2009; Miller 2014; Venojarvi 2013 |
| CRP | The mean CRP (mg/ml) in the intervention group was 0.05 lower (0.33 lower to 0.23 higher) | | - | 678 (4 RCTs) | ⨁⨁◯◯ LOW ^a,d^ | Included studies: Herder 2009; Lindhal 2009; Miller 2014; Yates 2010 |
| IL-6 | The mean IL6 (pg/ml) in the intervention group was 0.15 lower (0.25 lower to 0.04 lower) | | - | 775 (5 RCTs) | ⨁⨁◯◯ LOW ^a,d^ | Included studies: Gokulkrishna 2017; Herder 2009; Miller 2014; Venojarvi 2013; Yates 2010 |
| TNF-α | The mean TNF-α (pg/ml) in the intervention group is 0.67 higher (2.56 lower to 3.89 higher) | | - | 295 (3 RCTs) | ⨁◯◯◯ VERY LOW ^a,b,c^ | Included studies: Gokulkrishna 2017; Miller 2014; Venojarvi 2013 |
| ***The risk in the intervention group** (and its 95% confidence interval) is based on the assumed risk in the comparison group and the **relative effect** of the intervention (and its 95% CI).   **CI:** Confidence interval; **MD:** Mean difference | | | | | | |
| **GRADE Working Group grades of evidence** **High certainty:** We are very confident that the true effect lies close to that of the estimate of the effect **Moderate certainty:** We are moderately confident in the effect estimate: The true effect is likely to be close to the estimate of the effect, but there is a possibility that it is substantially different **Low certainty:** Our confidence in the effect estimate is limited: The true effect may be substantially different from the estimate of the effect **Very low certainty:** We have very little confidence in the effect estimate: The true effect is likely to be substantially different from the estimate of effect | | | | | | |

#### Explanations

a. Downgraded by one for risk of bias: Allocation concealment, selective reporting and attrition was a concern and unclear in the included studies.

b. Downgraded by one for Inconsistency due to considerable overall heterogeneity which could not be explained by sub-group analysis.

c. Downgraded by one for Imprecision for wide confidence interval and small sample size.

d. Downgraded by one for wide confidence interval

| **Physical activity with or without dietary or lifestyle modification compared to no intervention for Prediabetes** | | | | | | |
| --- | --- | --- | --- | --- | --- | --- |
| **Patient or population**: Prediabetes  **Setting**: China  **Intervention**: Physical activity with or without dietary or lifestyle modification  **Comparison**: No intervention | | | | | | |
| Outcomes | **Anticipated absolute effects^*^** (95% CI) | | Relative effect (95% CI) | № of participants  (studies) | Certainty of the evidence (GRADE) | Comments |
|  | **Risk with no physical activity** | **Risk with Physical activity with or without dietary or lifestyle modification** |  |  |  |  |
| Adiponectin (µg/ml) | The mean adiponectin (µg/ml) in the intervention group was 1.3 higher (0.57 lower to 3.17 higher) | | - | 61 (1 RCT) | ⨁◯◯◯ VERY LOW ^a,b^ | Included study: Liu 2017 |
| Leptin (ng/ml) | The mean leptin (ng/ml) in the intervention group was 2.08lower (3.87 lower to 0.29 lower) | | - | 61 (1 RCT) | ⨁◯◯◯ VERY LOW ^a,b^ | Included study: Liu 2017 |
| ***The risk in the intervention group** (and its 95% confidence interval) is based on the assumed risk in the comparison group and the **relative effect** of the intervention (and its 95% CI).   **CI:** Confidence interval; **MD:** Mean difference | | | | | | |
| **GRADE Working Group grades of evidence** **High certainty:** We are very confident that the true effect lies close to that of the estimate of the effect **Moderate certainty:** We are moderately confident in the effect estimate: The true effect is likely to be close to the estimate of the effect, but there is a possibility that it is substantially different **Low certainty:** Our confidence in the effect estimate is limited: The true effect may be substantially different from the estimate of the effect **Very low certainty:** We have very little confidence in the effect estimate: The true effect is likely to be substantially different from the estimate of effect | | | | | | |

Summary of findings table 2: Physical activity with or without dietary or lifestyle modification compared to no intervention for Prediabetes (Comparison 2)

#### Explanations

a. Downgraded by one for risk of bias for randomization, selective reporting and attrition was a concern and unclear in the included studies.

b. Downgraded by one for Imprecision for wide confidence interval and small sample size.
